# 

**Published:** 1949-07

**Authors:** 


					PUBLISHED UNDER THE AUSPICES OF THE BRISTOL MEDICO-CHIRURGICAL SOCIETY
THE BRISTOL
MEDICO - GHIRURGICAL
JOURNAL
A Journal of the Medical Sciences for the
WEST OF ENGLAND
AND SOUTH WALES
Editor:
E. WATSON-WILLIAMS, M.C., M.D., Ch.M., F.R.C.S.
with whom are associated
G. E. F. SUTTON, M.C., M.D., F.R.C.P.
J. M. NAISH, M.D., M.R.C.P.
N. S. CRAIG, M.C., M.D., Assistant Editor.
Local Correspondents ;
Bath?A. D. BATEMAN, O.B.E., F.R.C.S.
Bournemouth?A. MACKEZIE ROSS, M.D.
Cardiff?J. D. SPILLANE, M.D., M.R.C.P.
Exeter?Dr. L. N. JACKSON, M.C. Gloucester?Dr. R. F. JARRETT, M.B., M.R.C.P,
Newport?J. L. D. WILLIAMS, M.B., F.R.C.S. Plymouth?Dr. C. R. CROFT.
Taunton?Dr. M. McALLEY, D.M.R.
Torquay?Dr. A. ROBINSON THOMAS, D.M.R.E.
Truro?Dr. F. D. M. HOCKING, F.R.LC. Yeovil?J. A. VERE NICOLL, F.R.C.S.
BRISTol
J. W. ARROWSMITH LTD., QUAY STREET AND SMALL STREET
[Lj^LXVl. No. 239. Price: FOUR SHILLINGS net.
JULY * 1949

				

